# Estimating Utility Values for Health States of Nigerian Individuals with Stroke or Epilepsy Using the SF-36: A Brief Report on the Results of a Cross-Sectional Survey

**DOI:** 10.1177/23814683241266193

**Published:** 2024-08-02

**Authors:** T. Gebrye, C. O. Akosile, E. C. Okoye, U. V. Okoli, F. Fatoye

**Affiliations:** Manchester Metropolitan University, Manchester, LAN, UK; Nnamdi Azikiwe University, Nnewe, Nigeria; Nnamdi Azikiwe University, Nnewe, Nigeria; Nnamdi Azikiwe University, Nnewe, Nigeria; Manchester Metropolitan University, Manchester, LAN, UK; Lifestyle Diseases, Faculty of Health Sciences, North-West University, South Africa

**Keywords:** epilepsy, health status, quality-adjusted life-year, SF-36, SF-6D, stroke

## Abstract

**Background.** Stroke and epilepsy are the most common neurologic conditions affecting individuals. The Short Form Six-Dimension Health Index (SF-6D) is a preference-based measure of health developed to estimate utility values from the SF-36. This study estimated utility values for health states of Nigerian individuals with stroke or epilepsy using the SF-36. **Methods.** SF-36 responses from 125 and 69 individuals with stroke and persons with epilepsy, respectively, were transformed into health state utility values using the SF-6D algorithm. The Excel program developed by Brazier and colleagues was used to generate the SF-6D utility score estimated using a set of parametric preference weights. The health state utility values were determined using ordinal health state and standard gamble valuation techniques. **Results.** Mean (*s*) ages of the stroke and epilepsy participants were 63.1 (11) and 39.6 (16) y, respectively. The mean (*s*) utility scores for stroke and epilepsy were 0.52 (0.10) and 0.65 (0.1) for standard gamble and 0.48 (0.13) and 0.68 (0.11), respectively, using the ordinal health state paradigm. The mean (*s*) utility of stroke (female = 0.46 [0.15]; male = 0.50 [0.12]) and epilepsy (female = 0.65 [0.13], male = 0.69 [0.11]) participants were reported. The mean (*s*) annual episodes of seizure was 18.7 (39). **Conclusions.** To our knowledge, this is the first study to suggest that females with stroke and those with epilepsy considered their health to be poorer than that of their male counterparts. The significance of our findings is that they may be helpful for researchers, policy makers, and clinicians by providing input into economic evaluations to facilitate resource allocation for stroke survivors and people living with epilepsy to improve their health outcomes and reduce the huge burden associated with the conditions.

**Highlight:**

- We estimated a health state utility value for stroke and epilepsy to aid researchers and public health policy makers in conducting health economic analysis and outcomes research.

Neurologic disorders affect the brain and the nerves of the human body; they include stroke, Alzheimer’s disease, Parkinson’s disease, epilepsy, autism, migraine, cerebral palsy, and multiple sclerosis.^1,2^ Neurologic disorders account for more than 20% of the worldwide disease burden, and the greater majority of people affected with this type of disease are present in Africa.^3^ It has been predicted that by 2030, about 80% of all strokes will occur in low- and middle-income countries (LMICs), including Nigeria.^4^ Epilepsy is among the top 10 causes of disability in LMICs.^5^ In a study examining the scope of the burden of neurologic disease in Nigeria, the authors reported that of a total of 3,175 cases, 48.7% were neurologic disorders, among which epilepsy was the most common neurologic diagnosis, followed by stroke.^6^ Stroke is associated with a significant social and economic burden to individuals and society. The average annual cost per person with epilepsy in 2019 ranged from $204 in low-income countries to $11,432 in high-income countries.^7^

Depending on the type of neurologic disorder, its risk factors can include early exposure to chlamydia pneumonia, living in an area in which sun exposure is less frequent, smoking, diabetes, family history of stroke or heart disease, having a family history of Parkinson’s disease, and possessing certain genetic mutations.^8^ Stroke and epilepsy reduce active life expectancy.^9^ A study that measured the associations between strokes throughout older life and active life expectancy in African American and White women and men demonstrated that African American and White women were disabled for about two-thirds of life after stroke; results for men were 61.8% for African Americans and 37.2% for Whites.^10^

A prospective population-based study with a cohort of patients with newly diagnosed epilepsy in the United Kingdom found a slight decrease in lifetime expectancy compared with the general population.^11^ However, premature mortality is not the only consequence of stroke and epilepsy; both conditions also affect the health-related quality of life (HRQoL) of individuals.^12,13^ Measuring the health status of individuals is important because health status is considered in any equation that defines the benefits or effectiveness of interventions and is used to evaluate health care in terms of patient outcomes, to describe population health levels, and to study changes in health over time.^14^

Utility-based methods are used by health economists to combine the quality and quantity of life into a single measure such as quality-adjusted life-years (QALYs).^15^ People often use the terms *utility* and *preference* interchangeably.^16^*Preference* is a term used to describe the desirability of a set of outcomes measured using health status instruments such as the Short Form (SF)–12 and SF-36.^17^ On the other hand, utilities are a measure of the preference that an individual gives a particular health state with a number between 0 (death) and 1 (perfect health).^18^ Under conditions of uncertainty, utility-based methods are also used to elicit preferences through techniques such as the standard gamble (SG).^19^ To measure health utilities using the SG, it is necessary that the health state of interest is defined, the health states are assessed by individuals, and a value is placed on each of the health states.^17^ However, it is accepted that there is a significant limitation with the SG,^20^ and this could be minimized by the role of ordinal data in health state valuation.^21^ Ordinal health state valuation methods are important with ordering preferences of 2 or more alternatives without directly establishing the degree of preference of one alternative over the other.^22^ Overall, ordinal data have the potential to provide useful insights into community health state preferences, provided the differences in the information content between SG and ranking exercise.^23^

A few studies have examined the relationship between epilepsy and stroke and generic quality-of-life (QoL) instruments such as the Short Form 36 Health Survey Questionnaire (SF-36).^12,14,24–27^ The SF-36 is used to indicate the health status of a specific population across 8 separate dimensions: physical functioning, role–physical, bodily pain, general health, vitality, social functioning, role–emotional, and mental health.^28^ Scores for the SF-36 can be calculated using a range from 0 to 100, with higher scores indicating a better health state. However, the health state utility values used to weight the QALYs are obtained from the Short Form 6-dimension (SF-6D), a summary of a preference-based measure of health derived from the SF-36.

The health care system in Nigeria is characterized by a shortage of drugs and medical supplies.^29^ It is understood that efficient allocation of health care resources can enhance service delivery, improve access within existing budgets,^30^ and improve health outcomes. However, limited evidence of economic evaluation and its poor methodological quality in Africa, including Nigeria, have contributed challenges to allocation of resources in health care.^31^ Further, none of the existing studies^12,14,24–27^ were based on utility values using the SF- 6D,^20^ a summary preference-based measure of health derived from the SF-36. Therefore, the findings of these studies cannot be used to inform cost-effectiveness analysis of interventions for patients with these conditions. However, the SF-6D utility indexes could provide a means for using the SF-36 health surveys in economic evaluation by estimating a preference-based single index measure for health from these data. In the current study, we explored utilities estimated using the SF-6D, which could be used to obtain the quality adjustment weight required to calculate the QALYs in health economic models.^32^ The way the benefits of health care are measured and valued in economic evaluation is important, and QALYs are used to assess the effectiveness that is comparable across health care interventions. Thus, this study aimed to estimate the health utilities using data from a preference-based SF-36 for stroke survivors and people living with epilepsy.

## Methods

### Sample Description

This is a secondary analysis of data from previous studies on different populations of persons living with epilepsy (PLWE) and stroke survivors. Both the epilepsy and stroke studies were cross-sectional surveys involving adults (18 y and older) of PLWE and stroke survivors. PLWE were recruited from 3 purposively selected specialized clinics (RISE Clinic at Adazi-Ani, Nnamdi Azikiwe University Teaching Hospital at Ukpo, and Neuropsychiatric Hospital Nawfia) in southeastern Nigeria. Stroke survivors were also consecutively recruited from 7 conveniently selected tertiary hospitals from the 5 southeastern states in Nigeria. Stroke survivors were recruited if they lived in the community for at least 1 mo after discharge from the hospital. Stroke survivors who were not well oriented in time, place, or person, as ascertained by the clinicians taking care of the survivors, and stroke survivors who had any other conditions that might also cause disability, such as amputation, severe osteoarthritis, and rheumatoid arthritis, were excluded from the study.

The Ethics Committee of Nnamdi Azikiwe University Teaching Hospital, Nnewi, gave approval for the study. Permission was sought and obtained from the management of the various clinics before commencement of data collection. Both participants with stroke and epilepsy gave their informed consents either verbally or in written form after the procedure of the study has been duly explained to them. The few who gave verbal consent were required to thumbprint the informed consent form in the presence of their caregivers because of low education or because the dominant hand was affected by stroke.

### Variable Definitions

Utility values were used to estimate the reduction of HRQoL due to stroke and epilepsy. An algorithm developed by Brazier and colleagues^20^ was used to calculate utility values using the SF-6D, which is derived from the SF-36, into 6 dimensions such as physical functioning, role limitations, social functioning, pain, mental health, and vitality. On each of the 6 domains, respondents can be classified on any of 4 to 6 levels of functioning or limitations.^33^ The algorithm enabled us to estimate the SF-6D utility indexes that provide a means for using the SF-36 health surveys. The Excel program produces utility scores for the SF-6D from a sample of 69 and 125 individuals with epilepsy and stroke survivors, using ordinal and SG health state valuation models.^20^ In this study, we reported findings using an ordinal valuation technique that are comparable to the estimates produced using SG data.^20,22^ The ordinal preference method is based on a conditional regression estimated on rank data, which is rescaled to the 0 to 1 scale by dividing all coefficients for dimensions of health by the coefficient for the “dead” health state, as described in McCabe et al.^22^ The SF-6D utility score that was converted from SF-36 is useful to drive a QALY score for use in economic evaluation studies.

Information on sociodemographic (age, gender, location, marital status, occupational status, and highest educational attainment) and clinical (age at onset of epilepsy, episodes of seizure, comorbidities, presence of other therapies, and usage of assistive devices) variables of the participants were obtained through interviews. The sociodemographic variables (age, gender, education level, marital status, previous and current occupation) and the clinical variables of the stroke survivors (side of weakness, ambulation and poststroke duration) were also recorded.

### Statistical Analysis

Statistical analysis was undertaken using SPSS software (version 28.0.1.1; IBM SPS Statistics). For the utilities calculated using the Excel program developed by Brazier and colleagues, a descriptive analysis that included the use of percentages, frequencies, means, and ranges were used to describe the study sample.

## Results

The study sample mean (*s*) ages for stroke survivors and patients with epilepsy was 63.1 (11) and 39.6 (16) y, respectively. Seventy-nine percent of patients with stroke were married, whereas 59% of the patients with epilepsy were single. The highest percentage of patients with stroke (62.5%) and patients with epilepsy (39.1%) were employed and unemployed, respectively. Secondary and primary levels of education were achieved by 42.4% of the stroke survivors and 37.7% of patients with epilepsy. The mean age of onset of patients with epilepsy was 20.1 y. The mean poststroke duration in patients with stroke was reported as 22.3 mo (Table 1).

**Table 1:** Demographic Characteristics of the Study Sample

	Study Sample					
	Stroke			Epilepsy		
	M (*n* = 53)	F (*n* = 72)	Total (*n* = 125)	M (*n* = 48)	F (*n* = 21)	Total (*n* = 69)
Age, $\bar{x}$ (*s*)	62.5 (9.7)	63.5 (12.1)	63.1 (11)	39.8 (16.2)	39.3 (15.9)	39.6 (16)
Marital status, *n* (%)						
Single	5 (9.4)	3 (4.2)	8 (6.5)	25 (36.2)	16 (23.2)	41 (59.4)
Married	45 (84.9)	53 (74.6)	98 (79.0)	22 (31.9)	4 (5.8)	26 (37.7)
Widow	3 (5.7)	15 (21.1)	18 (14.5)	1 (1.4)	1 (1.4)	2 (2.9)
Occupation						
Employed	11 (21.2)	16 (22.2)	27 (21.8)	16 (23.1)	7 (10.1)	23 (31.9)
Self-employed	7 (13.5)	11 (15.3)	18 (14.5)	21 (30.4)	6 (8.7)	27 (39.1)
Others/unemployed	34 (65.4)	45 (62.5)	79 (62.5)	11 (15.9)	8 (11.6)	19 (27.5)
Level of education						
None	0 (0)	1 (1.4)	1 (0.8)	9 (13)	3 (4.3)	13 (17.4)
Primary	7 (13.2)	12 (16.7)	19 (15.2)	17 (24.6)	9 (13)	26 (37.7)
Secondary	23 (43.4)	30 (41.7)	52 (42.4)	12 (17.4)	7 (10.1)	19 (25.5)
Tertiary	20 (37.7)	26 (36.1)	46 (36.8)	8 (11.6)	2 (2.9)	10 (14.5)
Others	3 (5.7)	3 (4.2)	6 (4.8)	2 (2.9)	0 (0.0)	2 (2.9)
Mean (*s*) age on set of epilepsy, y	NA	NA	NA	21.2 (19.9)	17.6 (16.2)	20.1 (18.8)
Annual episodes of seizure	NA	NA	NA	14.2 (19.7)	28.9 (63.9)	18.7 (38.9)
Poststroke duration, mo	25.1 (23.9)	20.4 (25.6)	22.3 (24.9)	NA	NA	NA
Body mass index, kg/m^2^	NA	NA	NA	27.8 (5)	27.2 (5.6)	27.6 (5.2)

F, female; M, male; N/A, not applicable.

### Health Status

Table 2 reports the basic descriptive statistics for the health utility values of stroke survivors and persons with epilepsy of the study. The SF-36 data were converted to SF-6D data using SG and ordinal health state. The mean utility scores for stroke survivors and patients with epilepsy were 0.52 and 0.65 for SG and 0.48 and 0.68, respectively, using the ordinal health state paradigm. The health utility score for males was higher than for females for both stroke survivors and persons with epilepsy.

**Table 2:** Estimated Mean (95% Confidence Interval) Values of Health State Utility for Stroke Survivors and Individuals with Epilepsy

Technique	Stroke			Epilepsy		
	M (*n* = 53)	F (*n* = 72)	Total (*n* = 125)	M (*n* = 48)	F (*n* = 21)	Total (*n* = 69)
Standard gamble	0.54 [0.51, 0.56]	0.51 [0.48, 0.53]	0.52 [0.50, 0.54]	0.66 [0.63, 0.69]	0.64 [0.59, 0.69]	0.65 [0.63, 0.68]
Ordinal health state	0.50 [0.46, 0.53]	0.46 [0.43, 0.50]	0.48 [0.45, 0.50]	0.69 [0.66, 0.72]	0.65 [0.59, 0.71]	0.68 [0.65, 0.71]

F, female; M, male.

**Figure 1:**
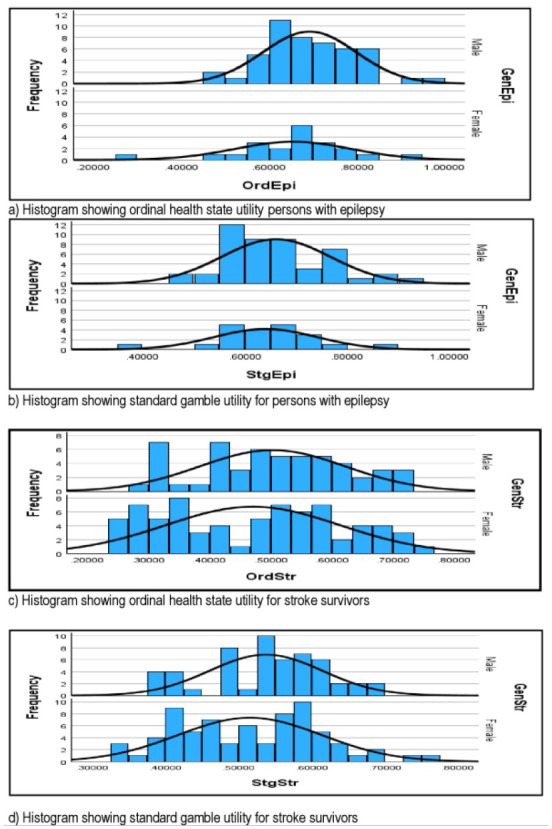
Histogram showing all the health utility values of stroke survivors and persons with epilepsy. (a) Histogram showing the ordinal health state utility for persons with epilepsy. (b) Histogram showing the standard gamble utility for persons with epilepsy. (c) Histogram showing the ordinal health state utility for stroke survivors. (d) Histogram showing the standard gamble utility for stroke survivors.

Figure 1 shows a histogram of all the health utility values of stroke survivors and persons with epilepsy of the study with estimated normal distributions.

## Discussion

To our knowledge, this is the first study to estimate the utility values for health states of patients with stroke and persons with epilepsy in Nigeria. The mean utility scores of the participants were assessed using the SF-6D. The SF-36 data were converted to SF-6D data using SG and ordinal health state. The findings of this study indicated that the mean utility scores in patients with stroke and epilepsy were 0.52 and 0.65 for SG and 0.48 and 0.68 using the ordinal health state paradigm, respectively.

We compared our findings with those of studies conducted in different countries.^34,35^ The mean utility values (0.584) of persons living with epilepsy in developed countries (the United Kingdom, France, Italy, Germany, and Spain) were higher than those of the current study.^34^ The pooled estimate of health utility values in stroke survivors for the SF-6D was reported as 0.70 (95% confidence interval [CI] 0.63–0.78; 2 studies),^35^ which is higher than the utility values for the Nigerian stroke survivors. The use of a different type of tool and the severity of the condition could be the reasons for the variation of health state utility values. For example, the study sample included in the study conducted in the United Kingdom, France, Italy, Germany, and Spain was people with less severe epilepsy.^34^ Previous studies also demonstrated the presence of variability in utilities between measurement techniques.^34,36^ Therefore, when measuring utilities, it is important to recognize that utility scores for the same health state could vary depending on which technique is used and the severity of the condition.

When undertaking economic evaluation in which QALY is the outcome of interest, it is important to have estimates of health utility values.^32^ Usually, health state utility value estimates are sourced from the published literature when there are no primary data. The results of the current study could thus be used by an economic analyst to undertake cost-effectiveness analyses of interventions for stroke survivors and individuals living with epilepsy. It is noted that preference measures such as the EQ-5D^37^ and the health utility index^38^ are alternatives to existing preference-based measures of health used in cost-effectiveness analysis. However, the SF-6D offers an improvement on these existing measures and the best method for modeling health state values.^39^ Therefore, it is crucial to ensure that the patient population (inclusion and exclusion criteria) and disease are well defined. If there are multiple published studies that estimate the health utility values of patients with stroke and persons with epilepsy, pooling the health utility value estimates across studies is vital, as it helps increase the accuracy of the results.^40^

A key strength of this study is that used the SF-36 item responses of the Nigerian individuals. The contributions of the variables such as annual episodes of seizure, poststroke duration, and body mass index were not included in the health utility values, so only a descriptive value was used for the study populations. The utility values are based on algorithms derived from the SF-36, and given the generic nature of this instrument, it may not capture important aspects of QoL (such as self-esteem) that may affect HRQoL. One must take caution in generalizing the findings of the present study due to the limited sample size. Although there are many states with various ethnicities, religions, and socioeconomic statuses in Nigeria, the areas surveyed included only some parts of the country, such as the southeastern states. If the study had included individuals from other areas, the evidence would have been stronger. A further limitation is the use of an existing algorithm for converting SF-36 data into SF-6D utilities. The key feature of this study is that it was able to use SF-36 item responses from the Nigerian population. While the current study provides estimates of utility, the utility estimates were based on Brazier’s utilities, in which the health states were elicited from respondents in the United Kingdom.^17^ Thus, further consideration of the choice of algorithm is important, as it could affect the validity of the reported utility. Nevertheless, this study provides some evidence that QoL in stroke survivors and individuals living with epilepsy could deteriorate when conditions are not well managed.

In conclusion, the results of this study provide a quantitative estimate suggesting that stroke survivors (0.48) and patients with epilepsy (0.68) could have health state utility values when measured using ordinal health state. The significance of our findings is that they may help inform resource allocation using QALYs to assess effectiveness that are comparable across health care interventions in stroke survivors and people living with epilepsy to improve health outcomes and reduce the huge burden associated with the conditions. On the other hand, the findings of our study could be used to obtain the quality adjustment weight required to calculate the QALYs in health economic models. Further, the magnitude of these estimates highlights the importance of capturing HRQoL as an outcome in future studies that evaluate policies designed to prevent or manage stroke and epilepsy.

## References

[1] Kanmounye US Abu-Bonsrah N Shlobin NA Djoutsop OM . The World Health Organization’s intersectoral global action plan on epilepsy and other neurological disorders 2022-2031. Neurosurgery. 2022;90(6):e201–3. 10.1227/neu.0000000000001976 35383690

[2] Gautam R Sharma M . Prevalence and diagnosis of neurological disorders using different deep learning techniques: a meta-analysis. J Med Syst. 2020;44(2):49. 31902041 10.1007/s10916-019-1519-7

[3] Burton KJ Allen S . A review of neurological disorders presenting at a paediatric neurology clinic and response to anticonvulsant therapy in Gambian children. Ann Trop Paediatr. 2003;23(2):139–43. 10.1179/027249303235002215 12803744

[4] Ezejimofor MC Chen YF Kandala NB , et al. Stroke survivors in low-and middle-income countries: a meta-analysis of prevalence and secular trends. J Neurol Sci. 2016;364:68–76. 27084220 10.1016/j.jns.2016.03.016

[5] Rathod S Pinninti N Irfan M , et al. Mental health service provision in low- and middle-income countries. Health Serv Insights. 2017;10:1178632917694350. 28469456 10.1177/1178632917694350 PMC5398308

[6] Onwuekwe IO Ezeala-Adikaibe B . Prevalence and distribution of neurological disease in a neurology clinic in Enugu, Nigeria. Ann Med Health Sci Res. 2011;1(1):63–8. PMC3507102 23209956

[7] Begley C Wagner RG Abraham A , et al. The global cost of epilepsy: a systematic review and extrapolation. Epilepsia. 2022;63(4):892–903. 35195894 10.1111/epi.17165

[8] Farooqui AA Farooqui T Panza F Frisardi V . Metabolic syndrome as a risk factor for neurological disorders. Cell Mol Life Sci. 2012;69:741–62. 10.1007/s00018-011-0840-1 PMC11115054 21997383

[9] Shavelle RM Brooks JC Strauss DJ Turner-Stokes L . Life expectancy after stroke based on age, sex, and rankin grade of disability: a synthesis. J Stroke Cerebrovasc Dis. 2019;28(12):104450. 31676160 10.1016/j.jstrokecerebrovasdis.2019.104450

[10] Laditka JN Laditka SB . Stroke and active life expectancy in the United States, 1999–2009. Disabil Health J. 2014;7(4):472–7. 10.1016/j.dhjo.2014.06.005 25096630

[11] Gaitatzis A Johnson AL Chadwick DW Shorvon SD Sander JW . Life expectancy in people with newly diagnosed epilepsy. Brain. 2004;127(11):2427–32. 10.1093/brain/awh267 15371287

[12] Okoye EC Okoro SC Akosile CO Onwuakagba IU Ihegihu EY Ihegihu CC . Informal caregivers’ well-being and care recipients’ quality of life and community reintegration—findings from a stroke survivor sample. Scand J Caring Sci. 2019;33(3):641–50. 10.1111/scs.12657 30734330

[13] Akosile CO Anomneze JU Okoye EC Adegoke BO Uwakwe R Okeke E . Quality of life, fatigue and seizure severity in people living with epilepsy in a selected Nigerian population. Seizure. 2021;84:1–5. 33248424 10.1016/j.seizure.2020.10.029

[14] Ware JE Jr Brook RH Davies AR Lohr KN . Choosing measures of health status for individuals in general populations. Am J Public Health. 1981;71(6):620–5. 10.2105/ajph.71.6.620 PMC1619843 7235100

[15] Yao H Hamilton HJ Geng L . A unified framework for utility-based measures for mining itemsets. In: Proceeding of ACM SIGKDD 2nd Workshop on Utility-Based Data Mining. Philadelphia, Pennsylvania, USA: Citeseer; 2006. p 28–37.

[16] Neumann PJ Goldie SJ Weinstein MC . Preference-based measures in economic evaluation in health care. Ann Rev Public Health. 2000;21(1):587–611. 10884966 10.1146/annurev.publhealth.21.1.587

[17] Brazier J Roberts J Deverill M . The estimation of a preference-based measure of health from the SF-36. J Health Econ. 2002;21(2):271–92. 10.1016/s0167-6296(01)00130-8 11939242

[18] Karimi M Brazier J . Health, health-related quality of life, and quality of life: what is the difference? Pharmacoeconomics. 2016;34:645–9. 10.1007/s40273-016-0389-9 26892973

[19] Whitehead SJ Ali S . Health outcomes in economic evaluation: the QALY and utilities. Br Med Bull. 2010;96(1):5–21. 21037243 10.1093/bmb/ldq033

[20] Brazier J Deverill M Green C . A review of the use of health status measures in economic evaluation. J Health Serv Res Policy. 1999;4(3):174–84. 10.1177/135581969900400310 10538884

[21] Kind P . Deriving cardinal scales from ordinal preference data: the analysis of time trade-off data using pairwise judgement models. Paper presented at: Health Economists Study Group; Brunel University, London; July 1996.

[22] McCabe C Brazier J Gilks P , et al. Using rank data to estimate health state utility models. J Health Econ. 2006;25(3):418–31. 10.1016/j.jhealeco.2005.07.008 16499981

[23] Arnoldner C Lin VY Honeder C Shipp D Nedzelski J Chen J . Ten-year health-related quality of life in cochlear implant recipients: prospective SF-36 data with SF-6D conversion. Laryngoscope. 2014;124(1):278–82. 10.1002/lary.24387 24122948

[24] Wang J Wang Y Wang LB Xu H Zhang XL . A comparison of quality of life in adolescents with epilepsy or asthma using the short-form health survey (SF-36). Epilepsy Res. 2012;101(1–2):157–65. 10.1016/j.eplepsyres.2012.03.017 22512895

[25] Birbeck GL Hays RD Cui X Vickrey BG . Seizure reduction and quality of life improvements in people with epilepsy. Epilepsia. 2002;43(5):535–8. 10.1046/j.1528-1157.2002.32201.x 12027916

[26] Mrabet H Mrabet A Zouari B Ghachem R . Health-related quality of life of people with epilepsy compared with a general reference population: a Tunisian study. Epilepsia. 2004;45(7):838–43. 10.1111/j.0013-9580.2004.56903.x 15230710

[27] Almborg AH Berg S . Quality of life among Swedish patients after stroke: psychometric evaluation of SF-36. J Rehabil Med. 2009;41(1):48–53. 19197569 10.2340/16501977-0287

[28] Burholt V Nash P . Short Form 36 (SF-36) health survey questionnaire: normative data for Wales. J Public Health. 2011;33(4):587–603. 10.1093/pubmed/fdr006 21307049

[29] Salisu S Mustafa MW Olatomiwa L Mohammed OO . Assessment of technical and economic feasibility for a hybrid PV-wind-diesel-battery energy system in a remote community of north central Nigeria. Alexandria Eng J. 2019;58(4):1103–18.

[30] Neumann PJ Rosen AB Weinstein MC . Medicare and cost-effectiveness analysis. N Eng J Med. 2005;353(14):1516. 10.1056/NEJMsb050564 16207857

[31] Panzer AD Emerson JG D’Cruz B , et al. Growth and capacity for cost-effectiveness analysis in Africa. Health Econ. 2020;29(8):945–54. 10.1002/hec.4029 PMC7383734 32412153

[32] Brazier J Ara R Rowen D Chevrou-Severac H . A review of generic preference-based measures for use in cost-effectiveness models. Pharmacoeconomics. 2017;35(suppl 1):21–31. 29052157 10.1007/s40273-017-0545-x

[33] O’Brien BJ Spath M Blackhouse G Severens JL Dorian P Brazier J . A view from the bridge: agreement between the SF-6D utility algorithm and the health utilities index. Health Econ. 2003;12(11):975–81. 10.1002/hec.789 14601159

[34] Flint I Medjedovic J Drogon O’Flaherty E , et al. Mapping analysis to predict SF-6D utilities from health outcomes in people with focal epilepsy. Eur J Health Econ. 2023;24(7):1061–72. 10.1007/s10198-022-01519-w 36260149

[35] Joundi RA Adekanye J Leung AA , et al. Health state utility values in people with stroke: a systematic review and meta-analysis. J Am Heart Assoc. 2022;11(13):e024296. 10.1161/JAHA.121.024296 PMC9333363 35730598

[36] Bozzani FM Alavi Y Jofre-Bonet M Kuper H . A comparison of the sensitivity of EQ-5D, SF-6D and TTO utility values to changes in vision and perceived visual function in patients with primary open-angle glaucoma. BMC Ophthalmol. 2012;12(1):1–9. 22909264 10.1186/1471-2415-12-43 PMC3552875

[37] Brooks R ; EuroQol Group. EuroQol: the current state of play. Health Policy. 1996;37(1):53–72. 10158943 10.1016/0168-8510(96)00822-6

[38] Torrance GW Furlong W Feeny D Boyle M . Multi-attribute preference functions: health utilities index. Pharmacoeconomics. 1995;7:503–20. 10.2165/00019053-199507060-00005 10155336

[39] Brazier J Roberts J Deverill M . The estimation of a preferencebased measure of health from the SF-36. J Health Econ. 2002;21(2):271–92. 10.1016/s0167-6296(01)00130-8 11939242

[40] Sena ES Currie GL McCann SK Macleod MR Howells DW . Systematic reviews and meta-analysis of preclinical studies: why perform them and how to appraise them critically. J Cereb Blood Flow Metabol. 2014;34(5):737–42. 10.1038/jcbfm.2014.28 PMC4013765 24549183

